# Therapeutic efficacy of hispidulin-loaded carboxymethyl cellulose nanoparticles against mammary carcinogenesis through ROS-mediated apoptosis and tissue remodelling

**DOI:** 10.3389/fonc.2026.1915182

**Published:** 2026-08-20

**Authors:** Vrushali Manoj Hadkar, Chinnadurai Immanuel Selvaraj

**Affiliations:** 1 Department of Biotechnology, School of Biosciences and Technology (SBST), Vellore Institute of Technology (VIT), Vellore, India; 2 Department of Genetics and Plant Breeding, VIT School of Agricultural Innovations and Advanced Learning (VAIAL), Vellore Institute of Technology (VIT), Vellore, India

**Keywords:** apoptosis, HIS-CMC NPs, immunohistochemistry, mammary carcinogenesis, oxidative stress, proliferation biomarkers

## Abstract

**Objective:**

To evaluate the apoptosis-modulating and therapeutic efficacy of hispidulin-loaded carboxymethyl cellulose nanoparticles (HIS-CMC NPs) in mammary carcinogenesis through *in vitro* and *in vivo* investigations.

**Methods:**

Apoptotic activity of HIS-CMC NPs was assessed in MCF-7 cells using cell viability, intracellular reactive oxygen species (ROS), DAPI, acridine orange/ethidium bromide (AO/EB), and Annexin-V/PI flow cytometry assays. Therapeutic efficacy was further examined in a DMBA-induced mammary carcinogenesis BALB/c mice model through haematological, biochemical, histopathological, immunohistochemical and oxidative stress analyses.

**Results:**

HIS-CMC NPs significantly reduced MCF-7 cell viability and exhibited a lower IC_50_ value (34.36 ± 1.66 μg/mL) than free hispidulin. Enhanced ROS generation, chromatin condensation, nuclear fragmentation, membrane destabilisation, and increased late apoptotic cell populations confirmed ROS-mediated apoptosis. *In vivo*, HIS-CMC NPs improved haematological and biochemical parameters, restored tissue architecture, downregulated *Ki67* and *Nrf2* expression, restored antioxidant enzyme activities, and reduced lipid peroxidation and lactate dehydrogenase levels.

**Conclusions:**

HIS-CMC NPs effectively promoted ROS-dependent apoptosis and attenuated oxidative stress in mammary carcinogenesis. The nanoformulation demonstrated enhanced therapeutic efficacy with reduced systemic toxicity, highlighting its potential as a promising nanotherapeutic strategy for breast cancer management. HIS-CMC NPs, mammary carcinogenesis, apoptosis, oxidative stress, proliferation biomarkers, immunohistochemistry.

## 1 Introduction

Cancer encompasses a complex array of diseases that pose a significant clinical challenge, impacting and claiming the lives of millions worldwide (1). Breast cancer remains one of the most prevalent forms of cancer in women, with a higher incidence and mortality rate. According to the latest GLOBOCAN 2024 estimates, approximately 2.43 million new breast cancer cases and 693,660 deaths were reported globally, accounting for 11.8% of all new cancer cases and 7.1% of all cancer-related deaths (2). Its incidence is projected to increase by 38% by 2050, particularly in low-HDI (Low-Human Development Index) regions with limited access to early diagnosis and treatment (3). Breast cancer is a heterogeneous disease classified into ER-positive, HER2-positive, and triple-negative (TNBC) subtypes based on ER, PR and HER2 expression (4, 5). Its development is influenced by multiple genetic, hormonal, environmental, and lifestyle factors, with oxidative stress and hormonal imbalance contributing to tumour progression (6). Among the molecular markers, estrogen receptor (ER) expression plays an important role in breast cancer diagnosis, prognosis, and therapeutic decision-making (7). Despite advances in screening and treatment, limited access to early diagnosis in many regions results in delayed detection, poor clinical outcomes, and increased mortality, highlighting the need for more effective therapeutic strategies (8, 9).

Conventional cancer treatments include surgery, radiotherapy, and chemotherapy. However, chemotherapy is often limited by non-specific drug delivery, systemic toxicity, multidrug resistance, and severe adverse effects (10). Cancer treatment mostly involves medications such as methotrexate, doxorubicin, celecoxib, 5-fluorouracil, paclitaxel and tamoxifen. The breast cancer initially shows a positive response to conventional chemotherapy. However, it ultimately develops resistance and metastasize during the later stages (5). One of the biggest obstacles is getting the medication to the target site and minimising toxicity to normal cells (11). Chemotherapeutic drugs commonly cause side effects such as fatigue, nausea, hair loss, weakened immune system and damage to healthy cells, including those in the digestive tract, bone marrow, and nervous system. These side effects can lead to anaemia, an increased risk of infections, mouth sores, and, in some instances, lasting damage to essential organs like the heart and nerves (12, 13). As an alternative, natural compounds from plants are gaining more attention as potential cancer treatments. This is mainly because they tend to be less toxic and can effectively target various pathways that contribute to tumour growth (14). Based on the numerous studies, plant-derived chemicals and associated products may offer the safest and most effective ways to treat various cancers (15).

Bioactive phytochemicals, including curcumin, resveratrol, epigallocatechin gallate, and sulforaphane, exhibit potent anticancer activities by inhibiting tumour growth and inducing apoptosis (16). Among these, flavonoids such as quercetin and icariin have demonstrated significant anticancer potential against breast cancer by modulating key signalling pathways and mitochondrial function (17, 18). Several flavonoids, including isorhamnetin, kaempferol, icariin, apigenin, and luteolin, have been shown to inhibit MCF-7 cell growth by inducing cell cycle arrest and apoptosis (19). One of the natural flavonoids, hispidulin, is reported for its notable anticancer activity, capable of inhibiting cancer cell growth and inducing apoptosis. These effects are mediated through ROS generation, ER stress activation and regulation of JAK2/STAT3 and PIM1 signalling pathways, resulting in reduced tumour progression and metastasis (20, 21). Hispidulin (4′,5,7-trihydroxy-6-methoxyflavone) is a bioactive flavone with a molecular weight of 300.26 g/mol and contains hydroxyl and methoxy functional groups responsible for its diverse pharmacological activities. However, its therapeutic application is restricted by poor aqueous solubility, limited bioavailability and stability issues (22). Thus, nanotechnology-based delivery systems overcome these challenges by improving drug solubility, tumour targeting, cellular uptake, and controlled drug release while reducing systemic toxicity (23, 24). Polymeric nanoparticles were selected over other nanocarriers such as micelles and liposomes due to their superior structural stability, efficient encapsulation, protection of the drug from degradation, and controlled release properties (25, 26). Additionally, the biodegradable nature and functional groups of carboxymethyl cellulose facilitate drug interaction and formulation stability, making it a suitable carrier to enhance the solubility, stability and therapeutic potential of hispidulin.

The polymeric NPs serve as effective carriers in cancer treatment, offering enhanced drug loading, biocompatibility and the ability to precisely control and target drug release, which improves therapeutic outcomes and reduces adverse effects (27). The carboxymethyl cellulose (CMC) as biopolymeric carrier are gaining attention as promising carriers in oncologic treatment with their compatible nature, biodegradability and capacity to deliver prolonged and targeted drug release (28, 29). In addition, CMC exhibits excellent viscosity-modifying and film-forming properties, which facilitate the fabrication of stable nanoparticulate systems. Its abundant hydroxyl and carboxyl functional groups provide versatile sites for chemical modification and interaction with therapeutic agents, making it an attractive polymer for advanced drug delivery applications (30, 31). Previously, we have synthesised hispidulin-loaded sodium carboxymethyl cellulose nanoparticles (HIS-CMC NPs) and evaluated their biological properties (32). The compound hispidulin was identified in the plant *Mundulea sericea* through LC-MS QTOF analysis. It has shown significant binding affinity for receptors linked to breast cancer based on the molecular docking study (32). The hispidulin was encapsulated into carboxymethyl cellulose nanoparticles (HIS-CMC NPs) to improve its therapeutic efficacy. The detailed physicochemical analysis revealed good encapsulation efficiency, appropriate particle size and regulated drug-release behaviour. The preliminary biological evaluations suggested enhanced biological performance of HIS-CMC NPs. These results demonstrated the potential of HIS-CMC NPs; however, thorough mechanistic validation and *in vivo* evaluation were not investigated.

Most research on anticancer effects of hispidulin has focused on the free compound, with investigations largely constrained to *in vitro* cell-based experiments evaluating cytotoxicity, apoptosis and molecular mechanisms in breast cancer cells (33–35). The novelty of this study is the use of carboxymethyl cellulose (CMC) NPs as hispidulin carrier. This gap emphasises the need for more investigation beyond initial *in vitro* data to assess the relative advantages and mechanisms of hispidulin-loaded CMC NPs administration for the treatment of mammary gland carcinogenesis. The purpose of this study was to determine whether nanoencapsulation of hispidulin using a biocompatible polymeric carrier could improve its anticancer efficacy and provide a more effective therapeutic strategy against mammary carcinogenesis in *in vivo*. Therefore, the present study aims to evaluate and compare the anticancer efficacy of free hispidulin and HIS-CMC NPs using *in vitro* assays on MCF-7 cells, and to further validate their therapeutic effect in a female BALB/c animal model. This work is the first of its kind, which represents a thorough and integrated comparison of HIS-CMC NPs and free HIS. It establishes a crucial connection between formulation design and translational anticancer efficacy by specifically examining the therapeutic superiority and mechanistic action of nanoencapsulated hispidulin. Thus, this work describes HIS-CMC NPs as a promising nanotherapeutic approach for the treatment of mammary carcinogenesis.

## 2 Materials and methods

### 2.1 Chemicals and reagents

Hispidulin - HIS (Cat. No. CFN99491; 98%) with average molecular weight = 300.3 g/mol was purchased from Chem Faces, Hubei, China. Trichloroacetic acid (TCA)–99%, Phosphate buffer saline (PBS) – 95.5%, Sulforhodamine B SRB – 75%, tris base – 99%, dichloro-dihydro-fluorescein diacetate (DCFH-DA) – 97%, 4′,6-diamidino-2-phenylindole (DAPI) – 98%, paraformaldehyde – 95%, acridine orange (AO) – 95%, ethidium bromide (EB) – 95%, Thio barbituric acid (TBA) – 98% and 6-hydroxy-2,5,7,8-tetramethylchroman-2-carboxylic acid (6-HD) – 98% were purchase from Sigma Aldrich, India. Diethylenetriaminepentaacetic acid (DTPA) – 99%, hydrogen peroxide (H_2_O_2_), potassium dichromate (K_2_Cr_2_O_7_) – 99.5%, glacial acetic acid (GAA) – 99.5%, 5,5′-Dithiobis-2-nitrobenzoic acid (DNTB) – 98%, NADH, sodium pyruvate – 99%, Bradford reagent and Sodium dodecyl sulfate (SDS) – 99% were obtained from SRL, India. Annexin V-FITC apoptosis detection kit was purchased from Thermo Fisher Scientific, USA. Dulbecco’s modified Eagle’s medium (DMEM) and trypsin–EDTA were acquired from Himedia, India.

Hispidulin-loaded carboxymethyl cellulose nanoparticles (HIS-CMC NPs) used in the present study were synthesised and comprehensively characterised in our previous work (32). The formulation exhibited appropriate physicochemical characteristics, high encapsulation efficiency, and sustained drug-release behaviour. The previously characterised HIS-CMC NPs were therefore employed for the *in vitro* apoptosis studies and *in vivo* evaluation against DMBA-induced mammary carcinogenesis described in the current investigation.

The MCF-7 (Job No. 2430/2023-24) cells were obtained from the National Centre for Cell Science - NCCS, Pune, India.

### 2.2 *In vitro* studies of HIS and HIS-CMC NPs

#### 2.2.1 SRB assay

The cytotoxicity of the HIS and HIS-CMC NPs against MCF-7 cells were assessed using the SRB assay (36). The cells were cultured in the six-well plates (2×10^5^ cells/well) and incubated for 24 hours at 37 °C and 5-6.5% CO_2_ (BST/UCI-42, Bionics Scientific, India). The cells were then exposed to (25-100 µg/mL) concentrations of HIS and HIS-CMC NPs for 24 hours. The blank CMC was used as negative control. The fixation of cells was done by adding cold TCA (10%) for two hours, followed by a PBS wash and staining with SRB (0.4%) dye for 20 min. After five rinses with 1% acetic acid, the cells were allowed to air dry for four hours. The treated and untreated cells were examined under a microscope (Leica Microsystems, Germany) for images. Additionally, Tris base (10 mM) was used to dissolve the bound SRB dye, and the absorbance was recorded at 510 nm through the microplate reader (CLARIOstar^®^ Plus, BMG LABTECH, Germany).


$$Cell\;viability\left(\%\right)=\frac{Optical\;density\;of\;sample}{Optical\;density\;of\;control}\times 100$$

#### 2.2.2 DCFH-DA staining assay

The MCF-7 cells were seeded onto six-well plates (2 × 10^5^ cells per well) and cultured for 24 hours. Following incubation, IC_50_ concentrations of HIS (62 µg) and HIS-CMC NPs (34 µg) were applied to the cells. The blank CMC was used as negative control. DCFH-DA stain was used to measure the ROS levels in MCF-7 cells. After two PBS washes, the cells were exposed to 500 μL of 10 μM DCFH-DA for 45 minutes at 37 °C in the dark conditions. Following incubation, unbound dye was removed from the MCF-7 cells by gently aspirating the DCFH-DA solution using a micropipette and washing them with PBS three times. The ROS was then examined using fluorescence microscopy (Wavelengths of excitation: 485 nm, emission: 535 nm) for treated and untreated MCF-7 cells (37).

#### 2.2.3 DAPI staining assay

The nuclear morphology of MCF-7 cells treated with HIS and HIS-CMC NPs was assessed through the DAPI staining technique. Six-well plates were used to culture the MCF-7 cells (2 × 10^5^ cells per well) for 24 h. Following the incubation period, the cells were treated with HIS and HIS-CMC NPs at IC_50_ concentrations and then incubated for 24 h. The cells were fixed with 3.7% paraformaldehyde for 15 minutes at room temperature and rinsed twice with PBS. Following a PBS wash, the cells were stained with 500 μL of 2.86 µM DAPI solution for 10 minutes at room temperature before being viewed for the nuclear characteristics under the fluorescence microscope (Wavelengths of excitation: 358 nm, emission: 461 nm) (38).

#### 2.2.4 AO/EB staining assay

The MCF-7 cells treated using HIS and HIS-CMC NPs were assessed for their cell death characteristics (apoptosis and necrosis) through the AO/EB staining method. The MCF-7 cells (2 × 10^5^ cells per well) were cultured in six-well plates for 24 hours. Following an incubation period, the cells were exposed to the IC_50_ concentrations of HIS and HIS-CMC NPs and incubated again for 24 hours. After an incubation period, the cells were washed with PBS twice and stained with 500 μL of AO/EB stain mix (350 µM and 300 µM). The cells were incubated for 10 minutes at room temperature in dark conditions. The apoptotic cells were observed under the fluorescence microscope (Wavelengths of excitation: 358 nm, emission: 461 nm) based on their morphological characteristics (39).

#### 2.2.5 Annexin V FITC assay

The percentage of apoptotic MCF-7 cells following treatment with HIS and HIS-CMC NPs was evaluated using an Annexin V-FITC apoptosis detection kit (Thermo Fisher Scientific, USA). The MCF-7 cells were cultured (20 × 10^4^ cells/flask) for 24 hours and treated with HIS and HIS-CMC NPs. After treatment, the cells were extracted using trypsin and washed with PBS by centrifuging (R-4C, REMI, India) for five minutes at 3000 rpm. The cells were then suspended in binding buffer. The propidium iodide and Annexin V were added to the cells and kept in the dark at room temperature. Using a flow cytometer (CytoFLEX S, V4-B2-Y4-R3, Beckman Coulter, US) and Novo Express 1.0.2 software, the fluorescence intensity of FITC and PI was quantified to determine the number of viable, early apoptotic and late apoptotic cells (40).

### 2.3 *In vivo* studies of HIS and HIS-CMC NPs

#### 2.3.1 Maintenance of animals

Experimental procedures involving animals received approval from the Institutional Animal Ethics Committee (Approval No. VIT/IAEC-26/17Apr/12) and were carried out in accordance with ARRIVE guidelines. We have used thirty healthy female BALB/c mice (*Mus musculus*) that were 6–7 weeks old and weighed 20–30 g, obtained from Biogen Laboratory Animal Facility (Anekal, Bangalore, India). They were housed in well-ventilated cages with standard light conditions (12 h with alternate day and night cycles) and temperature (23 ± 2 °C) in the CCSEA-registered animal facility. During the experiment, the animals were fed with the standard lab pellets (KVA-LLP, Pune, India) and had access to water *ad libitum*. The animals were weighed once a week and monitored every day to observe changes in their body weight and behavioural patterns.

#### 2.3.2 Experimental design

A total of thirty female BALB/c mice were allocated into five groups (n = 6 per group), of which four groups were subjected to mammary gland carcinogenesis induction. Group I: Normal control (vehicle corn oil through oral gavage); Group II: Disease control (DC) (DMBA 1 mg/kg through oral gavage); Group III: Treatment (HIS 10 mg/kg through i. p.); Group IV: Treatment (HIS-CMC NPs 10 mg/kg through i. p.) and Group V: Positive control (Cisplatin 5 mg/kg through i. p.). The mammary gland carcinogenesis was induced using the chemical DMBA, except for the normal control group. A dose of 1 mg/kg body weight of DMBA in 1 ml of corn was given to animals through oral gavage 6 times a week for 6 weeks (41). Treatments of HIS, HIS-CMC NPs and cisplatin were given through intraperitoneal injection twice a week for 16 weeks.

#### 2.3.3 Collection of blood and tissue samples

The blood samples (0.5 mL) were collected through cardiopuncture while animals were under anaesthetic conditions and transferred into tubes containing EDTA, which were stored at -80 °C for haematological and biochemical analysis. The mammary glands were dissected to observe the precancerous lesions, and other organs such as lungs, kidneys and liver were promptly dissected for each group of animals and rinsed in PBS. Half of the collected organs were preserved in 10% buffered formalin for histopathological examination. The remaining organs were stored in 50 mM phosphate buffer (pH 7.5) and preserved at −80 °C for subsequent biochemical studies.

#### 2.3.4 Histopathological and immunohistochemical analysis

After the processing of tissue samples (mammary glands, liver, kidney and lungs), the rotary microtome (Leica RM 2125 RTS, Leica Biosystems, Germany) was used to trim and segment the tissues at a size of 5 µm. The sections were placed on glass slides and left to dry overnight at 56 °C. Slides were rehydrated using graded ethanol, deparaffinized in xylene and then rinsed with distilled water. Filtered haematoxylin was used to stain the tissues, followed by a wash, 0.25% acid alcohol for differentiation, and eosin for counterstaining. After dehydration through ethanol and clearing in xylene, the slides were coated with cytoseal and examined under a light microscope (42). Mammary gland tissues were preserved in 10% formaldehyde and embedded in paraffin for immunohistochemistry. The antigen retrieval in sodium citrate buffer (pH 6.0) was applied to five-micron slices on poly-L-lysine-coated slides. The sections were blocked with 2% BSA, then incubated with ER-α primary antibody (1:200) for an entire night before being exposed to secondary antibody for 60 min at ambient temperature. The counterstain haematoxylin and 3,3′-diaminobenzidine was used to produce colour. At 400× magnification (LYNX LM-52–1711 Microscope, Lawrence & Mayo Pvt. Ltd, India), ER-positive cells were counted from ten randomly chosen fields, and protein expression was computed for each group (43).

### 2.4 Biochemical studies

#### 2.4.1 Preparation of tissue homogenate

The tissue homogenate was prepared by using the samples of mammary gland, lungs, liver and kidney tissues, which were stored at -80 °C. Tissue samples of each organ were weighed (1 g) and added to cold PBS (9 mL, pH 7.5). The sample was homogenised using a mortar and pestle in cold conditions. After centrifuging the homogenised sample for 15 min at 10,000 rpm, the supernatant was collected and kept at -80 °C for the biochemical studies (44).

#### 2.4.2 Malondialdehyde activity

Lipid peroxidation in the tissue samples (mammary gland tissues, liver, kidney and lungs) was estimated using the Thio barbituric acid reactive substances (TBARS) assay. The supernatant of the tissue (200 μl) was mixed with 100 μl of SDS, each separately. After adding 500 μl of 20% TCA to each tube, the mixture was vortexed. Following a fifteen-minute centrifugation at 2000 rpm, the clear supernatant was removed and transferred into separate tubes. Further, 250 μl of 0.8% TBA and 75 μl of EDTA were added. The tubes were kept in a water bath at 90 °C for 40 minutes, followed by vortexing. The MDA activity was measured spectrophotometrically (UV-VIS, UV-1280, Shimadzu, Japan) at a wavelength of 532 nm, and the results were calculated as nanomoles per milligram protein (nmol/mg protein) (45).

#### 2.4.3 Superoxide dismutase activity

The SOD activity was measured spectrophotometrically by monitoring the inhibition of substrate autoxidation caused by superoxide radicals. The tissue supernatant (200 µL) for each tissue was added with 15 μl of 1.6 mM 6-HD and 170 μl of 0.1 mM DTPA. The tubes with the reaction mixture were vortexed and incubated for 10 minutes at room temperature. Following the incubation period, the absorbance was measured at 492 nm using spectrophotometry. The SOD activity was represented as units per milligrams of protein (U/mg protein) (46).

#### 2.4.4 Glutathione reductase activity

The dithionitrobenzoic acid recycling method was used to measure reduced glutathione (GSH) levels in tissues (45). Briefly, 200 μL of tissue supernatant was combined with 50 μL of 1mM Ellman’s reagent (DNTB in 0.1 M PBS with pH 7.4). The reaction mixture tubes were incubated for five minutes at room temperature, and the absorbance was determined spectrophotometrically at 412 nm. The GSH activity was represented as units per milligram of protein (U/mg protein).

#### 2.4.5 Catalase activity

The dichromate method was used to assess catalase activity (47). Two hundred microlitres of tissue supernatant was combined with 600 µL of 6.0 mM PBS pH 7.4 and 200 µL of 30 mM H_2_O_2_. The dichromate reagent -5% potassium dichromate in glacial acetic acid (500 µL) was added to terminate the reaction. The reaction mixture was incubated for ten minutes at 37 °C in a water bath, and the absorbance at 620 nm was measured. The breakdown of H_2_O_2_ was used to calculate the catalase activity and expressed as units per milligram of protein (U/mg protein).

#### 2.4.6 Lactate dehydrogenase activity

Lactate dehydrogenase activity was measured to assess tissue damage and cellular integrity as an indicator of oxidative stress. The reaction mixture comprised 30 mM sodium pyruvate, 7.5 mM NADH and 100 µL of 0.1 M PBS (pH 7.4). A suitable volume of tissue supernatant (80 µL) was added to initiate the reaction after pre-incubation at 37 °C. The decrease in absorbance at 340 nm was monitored for three to five minutes. The LDH activity (U/mL) was computed (48).

### 2.5 Statistical analysis

The results obtained from the present study were analysed by using ANOVA. Data were computed for statistical analysis by using GraphPad Prism version 5.00 for Windows, GraphPad Software version 8, San Diego, CA, USA and compared with the control group. The data were represented as mean ± SD with *p < 0.05, **p < 0.01, ***p < 0.001 and ns – non-significant.

## 3 Results

### 3.1 Characterisation of HIS-CMC NPs

The physicochemical characteristics of HIS-CMC NPs were previously reported in our earlier study (32). The successful encapsulation of HIS within the CMC matrix was confirmed by UV–Vis, XRD, and FTIR analyses. UV–Vis spectra showed characteristic HIS absorption peaks at 216, 274, and 336 nm, with slight shifts, reduced intensity, and band broadening in HIS-CMC NPs, indicating HIS–CMC interactions. XRD analysis demonstrated sharp crystalline peaks for HIS, whereas HIS-CMC NPs exhibited broad diffraction patterns, suggesting reduced crystallinity after encapsulation. FTIR spectra confirmed the presence of characteristic functional groups of both HIS and CMC, with shifts in O–H and carboxylate bands indicating hydrogen bonding and drug–polymer interactions. These findings confirmed the successful incorporation of HIS into the CMC nanoparticulate system. Briefly, HIS-CMC NPs exhibited a hydrodynamic diameter of 243.3 nm with a low PDI value of 0.181 and a zeta potential of -39.1 mV, indicating uniform particle distribution and good colloidal stability. Morphological analysis by FESEM and HRTEM confirmed the nanoscale nature of the particles with an average size of approximately 160 nm and 150.35 nm, respectively. These optimised characteristics support the suitability of HIS-CMC NPs for subsequent biological evaluation. In the present study the UV-Vis analysis of CMC was performed (**Supplementary Figure 1**). CMC showed a characteristic absorption peak at 252 nm in the UV–Vis spectrum, which was attributed to the intrinsic absorption of the polymer structure (49).

### 3.2 *In vitro* studies of HIS and HIS-CMC NPs

#### 3.2.1 SRB assay

**Figure 1:**
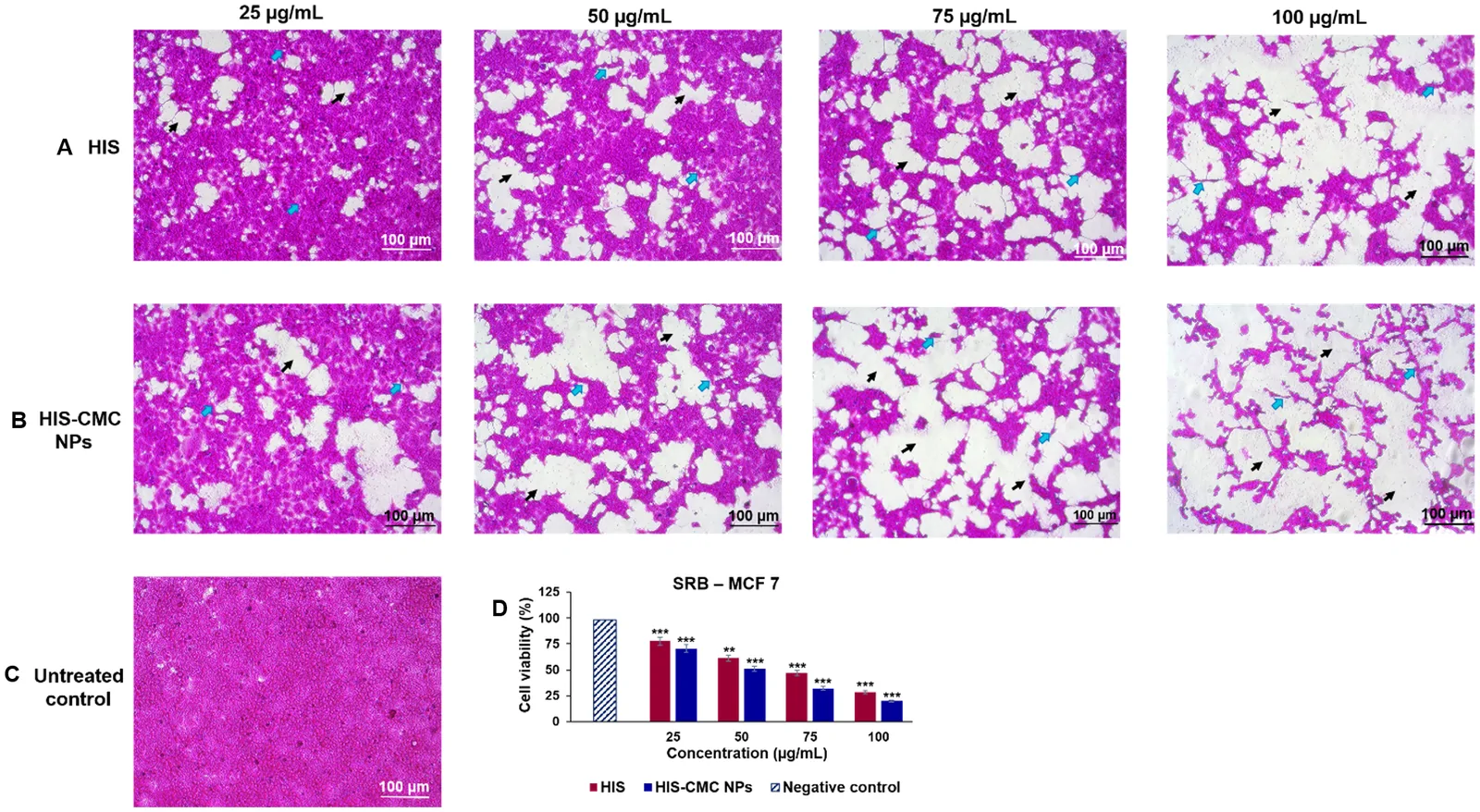
Sulforhodamine B assay showing the dose-dependent cytotoxic effect of HIS and HIS-CMC NPs on MCF-7 cells. Representative SRB-stained micrographs of cells treated for 24 h with **(A)** HIS and **(B)** HIS-CMC NPs at 25, 50, 75 and 100 µg/mL and **(C)** untreated control, demonstrating progressive loss of the adherent (black arrows), cell shrinkage (blue arrows), **(D)** Quantitative SRB cell-viability data expressed as percentage of reduced viability for both HIS and HIS-CMC NPs. Statistically significant differences were determined by Tukey’s multiple comparison tests for treated groups compared with the control (^*^p < 0.05, ^**^p < 0.01, ^***^p < 0.001). Data are represented as Mean ± SD (n = 3).

In this study, the SRB assay revealed a dose-dependent decrease in MCF-7 cell viability following treatment with HIS and HIS-CMC NPs compared to the control (**Figures 1A, B**). The increasing concentrations of HIS and HIS-CMC NPs (25–100 µg/mL) progressively reduce the extent and intensity of SRB staining, with large unstained or weakly stained areas reflecting loss of adherent cells and marked cytotoxicity. The partial disruption of the monolayer was observed at 25 and 50 µg/mL, while at 75 and 100 µg/mL, it displayed higher cell death and growth inhibition in comparison to the control (**Figure 1C**). The bar graph quantitatively confirmed the morphological observations, showing a significant, dose-dependent decline in percentage viability of MCF-7 cells treated with HIS and HIS-CMC NPs compared with the negative control (**Figure 1D**). The lowest cell viability was reported at 100 µg/mL, indicating strong antiproliferative effects. The MCF-7 cells, after being treated with HIS and HIS-CMC NPs, displayed 28.32 ± 1.86 and 19.79 ± 0.81 percentage of cell viability with IC_50_ values of 62.84 ± 1.79 and 34.36 ± 1.66 μg/mL, respectively. The HIS-CMC NPs retained comparatively higher inhibitory activity than HIS across the concentration range (25-100 µg/mL) because of enhanced cellular absorption, HIS protection against degradation and sustained intracellular release profile, which increased the cytotoxic effect of the NPs against MCF 7 cells. The contribution of the polymeric carrier, CMC was also assessed in MCF-7 cells. Treatment with CMC did not produce any noticeable cytotoxicity, and the cells retained normal morphology comparable to the untreated control (**Supplementary Figure 2**). In contrast, HIS and HIS-CMC NPs significantly reduced cell viability, indicating that the observed cytotoxicity was primarily due to hispidulin rather than the CMC polymer. The cell viability was considerably reduced by HIS-CMC NPs compared to free HIS at equivalent concentrations (*p* < 0.001), which is consistent with the IC_50_ value.

#### 3.2.2 DCFH-DA staining assay

**Figure 2:**
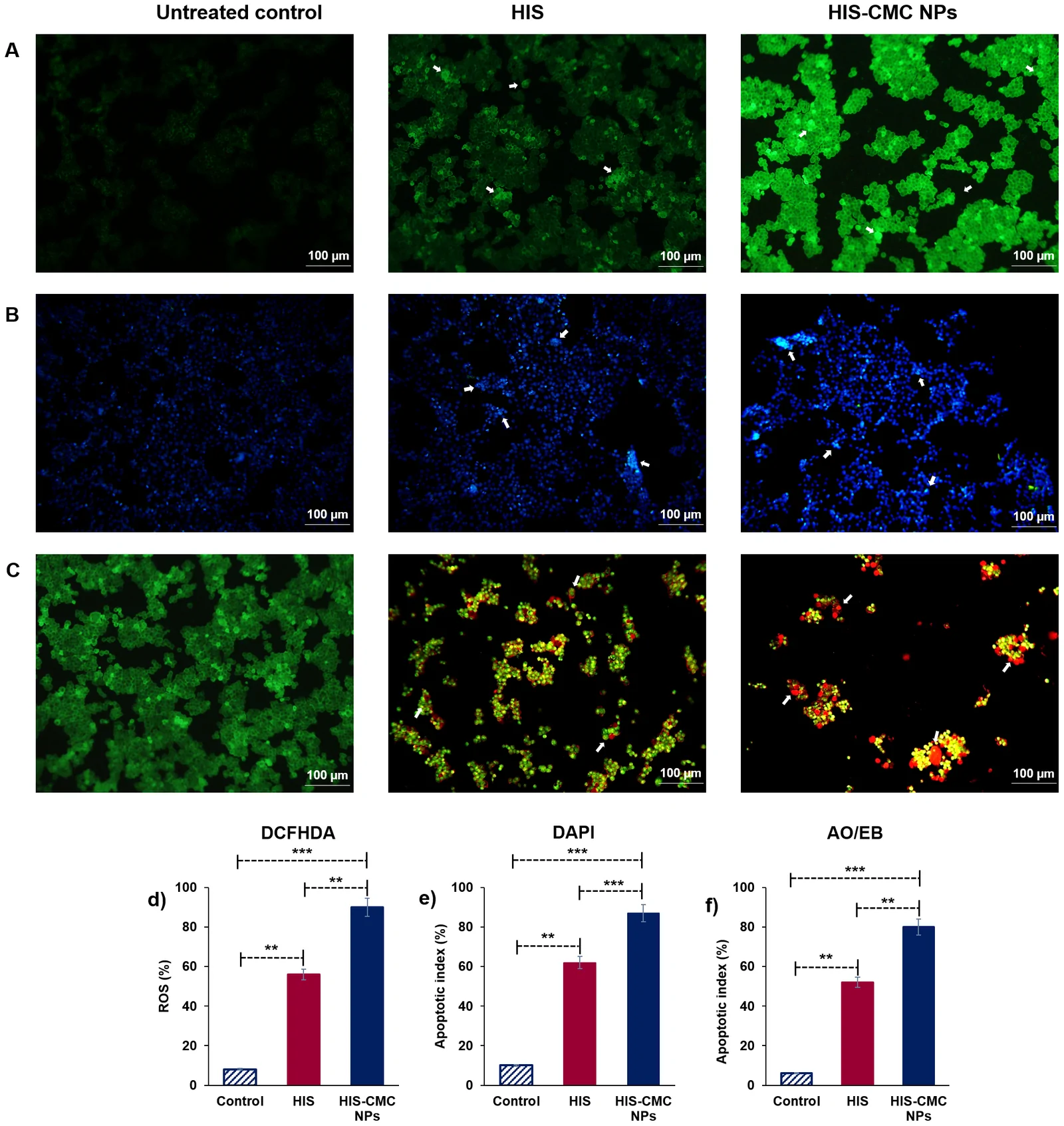
Qualitative and quantitative assessment of oxidative stress and apoptosis in MCF-7 cells after treatment with HIS and HIS-CMC NPs. **(A)** DCFH-DA assay showing intracellular ROS generation, **(B)** DAPI assay demonstrating nuclear morphological changes, **(C)** AO/EB dual assay revealing viable (green), early apoptotic (yellow), and late apoptotic (red) cells. Images were captured using a fluorescence microscope at 20x magnification with a scale bar of 100 µm. The bar graphs represent **(D)** quantification of ROS generation, **(E)** DAPI apoptotic index, and **(F)** AO/EB apoptotic percentage. Statistically significant differences were determined by Tukey’s multiple comparison tests for treated groups compared with the control ^(**^p < 0.01, ^***^p < 0.001). Data are represented as Mean ± SD (n = 3).

The present study demonstrated that HIS and HIS-CMC NPs markedly elevated the intracellular ROS levels in MCF-7 cells compared to the negative control. It was found that the MCF 7 cells treated with free HIS alone showed brighter punctuate green fluorescence, whereas cells treated with HIS-CMC NPs produced intense green fluorescence showing higher ROS generation than free HIS. The negligible green fluorescence indicating the basal ROS was observed in the untreated control cells, while a tremendous increase in the fluorescence intensity was visualised in cells treated with HIS-CMC NPs (**Figure 2A**). These findings are supported by the quantitative bar graph (**Figure 2D**), which displays a considerable increase in ROS percentage for HIS and a significant rise for HIS-CMC NPs. The significantly higher ROS levels in HIS-CMC NPs treated cells (90.34 ± 1.62%), as compared to HIS (56.67 ± 1.22%) and control, showed that HIS nanoencapsulation increased oxidative stress-mediated cytotoxicity in MCF-7 cells. Compared with the negative control, both HIS and HIS-CMC NPs significantly increased intracellular ROS generation (*p* < 0.001), with HIS-CMC NPs exhibiting a significant effect than free HIS (*p* < 0.001).

#### 3.2.3 DAPI staining assay

In MCF-7 cells, DAPI labelling showed treatment-dependent nuclear changes indicative of apoptosis. In the negative control, nuclei appeared uniform, spherical and faintly blue, indicating intact chromatin and negligible spontaneous apoptosis. The HIS-treated cells displayed several brilliantly luminous nuclei with chromatin condensation and disintegration, indicating the activation of apoptotic cell death (**Figure 2B**). The cells treated with HIS-CMC NPs exhibited an even higher proportion of intensely stained, condensed and fragmented nuclei, suggesting that nanoencapsulation of HIS enhanced its apoptosis-inducing potential with the increased ROS levels and reduced viability of MCF 7 cells. The observed apoptotic index for HIS and HIS-CMC NPs was 62.12 ± 0.46% and 87.66 ± 0.23%, respectively, while the untreated control displayed a negligible apoptotic index (**Figure 2E**). When compared to the negative control, MCF-7 cells treated with HIS and HIS-CMC NPs had a considerably higher apoptotic index (*p* < 0.001). The HIS-CMC NPs also demonstrated a highly significant elevation when compared to free HIS (*p<* 0.001), indicating significant pro-apoptotic efficacy of the NPs, according to quantitative analysis of apoptotic nuclei in MCF-7 cells.

#### 3.2.4 AO/EB staining assay

The free HIS treated cells exhibited more yellowish-orange and reddish nuclei, particularly those shown by arrows, which indicate early and late apoptotic alterations, such as chromatin fragmentation and condensation. The strong apoptosis induction by the NPs is confirmed by the MCF-7 cells treated with HIS-CMC NPs, which showed an increase in orange/red fluorescence together with dense clusters of apoptotic cells, indicating significant membrane breakdown and nuclear fragmentation compared to free HIS. The untreated MCF-7 control cells generally exhibited homogeneous green fluorescence, indicating healthy, viable nuclei with intact membranes (**Figure 2C**). The quantitative bar graph (**Figure 2F**) further supports these findings by demonstrating a substantial increase in the apoptotic index following treatment with HIS-CMC NPs (80.00 ± 0.31%), followed by free HIS (52.16 ± 0.22%), with both treatments showing a significant increase compared with the untreated control. The apoptotic index was statistically significant for both HIS and HIS-CMC NPs than for the negative control (*p* < 0.001). The HIS-CMC NPs also demonstrated a statistically significant elevation compared to HIS alone (*p* < 0.001), highlighting higher apoptotic efficacy of the HIS-CMC NPs.

Similarly, CMC induced negligible intracellular ROS generation, nuclear morphological alterations, and apoptotic cell death, exhibiting fluorescence patterns comparable to those of the untreated control (**Supplementary Figure 3**). In contrast, HIS and particularly HIS-CMC NPs markedly increased ROS production, induced nuclear condensation and fragmentation, and promoted apoptotic cell death, confirming that the observed cellular responses were predominantly due to the therapeutic action of hispidulin.

#### 3.2.5 Annexin V/PI assay

**Figure 3:**
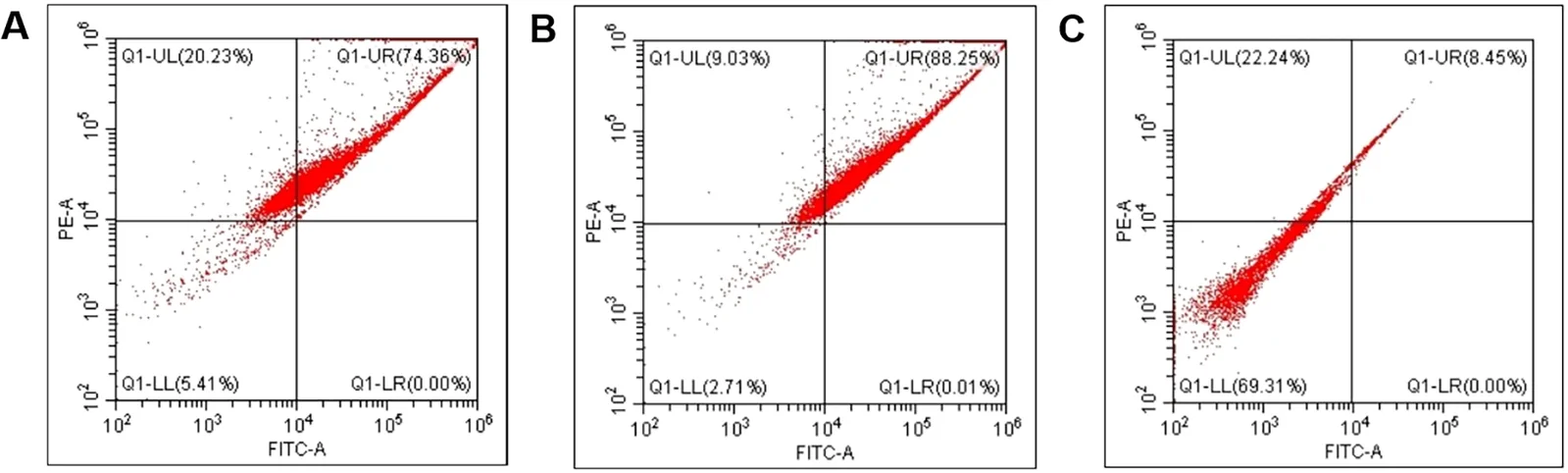
Annexin-V/PI flow cytometry analysis of apoptosis in MCF-7 cells treated with **(A)** HIS, **(B)** HIS-CMC NPs and **(C)** control for 24 h. Dot plots show forward/side scatter gating and Annexin-V (FITC) versus PI (PE) quadrants: Early apoptotic cells are shown in the lower-right panel, while late apoptotic cells are shown in the upper-right panel. Data are representative of three independent experiments. Quantification (Mean ± SD) of viable, early and late apoptotic percentages is shown.

Annexin-V/PI flow cytometry was utilised to quantify apoptosis in MCF-7 cells after 24h treatment. HIS treatment increased the proportion of Annexin-V–positive cells, with elevations in both early (Annexin-V+/PI−) and late apoptotic (Annexin-V+/PI+) fractions (**Figure 3A**). In comparison to free HIS and control, HIS-CMC NPs generated the highest apoptotic response, resulting in a notable decrease in viable cells and a considerably greater percentage of late apoptotic cells (**Figure 3B**). The control cells displayed a greater number of events in the viable quadrant (Annexin-V−/PI−), indicating reduced spontaneous apoptosis (**Figure 3C**). The Annexin-V data demonstrated that free HIS promoted programmed cell death in MCF-7 cells, and that CMC nanoparticle encapsulation considerably increased the effect. The increase in Annexin-V+/PI+ cells after HIS-CMC treatment shows progression from early apoptosis to late apoptosis/secondary necrosis, possibly demonstrating improved cellular absorption and long-term intracellular HIS release from the nanoparticle formulation. These findings are consistent with our complementary assays (DCFH-DA, DAPI, AO/EB), which indicated enhanced ROS production, mitochondrial depolarisation, and nuclear condensation in HIS-CMC treated cells, indicating intrinsic (mitochondria-mediated) apoptosis.

### 3.3 *In vivo* studies of HIS and HIS-CMC NPs

#### 3.3.1 Body weight and growth rate in animals

The initial body weights of all experimental groups at 6 weeks of age were comparable, suggesting that randomisation was successful before the induction of mammary carcinogenesis (**Table 1**). Group I (normal control) showed normal physiological growth and weight gain over the course of the 16-week experiment, and their final body weight was 42.52 ± 0.68 g. Conversely, the group II (disease control) showed a significant reduction in final body weight, which was 39.13 ± 0.74 g (*p<* 0.01) compared to the group I, suggesting a systemic burden linked to exposure to carcinogen DMBA. Group III treated with HIS exhibited a statistically significant (*p* < 0.05) increase in body weight (41.08 ± 0.59 g) as compared to group I. Notably, animals treated with HIS-CMC NPs (Group IV) showed a favourable growth pattern, reaching a final body weight of 43.62 ± 0.63 g (*p* < 0.01), indicating that the nano-formulation was biocompatible. Cisplatin treated animals (Group V) had the lowest body weight of 35.46 ± 0.71 g (*p* < 0.01) compared to the negative control, consistent with the known systemic toxicity of conventional chemotherapy.

**Table 1:** Body weight parameter of experimental animals during the study period.

S. no.	Treatment	Initial body weight (g) (6 weeks old)	Final body weight (g) (16 weeks old)	Growth rate (%)
1.	Group I: Normal control (vehicle corn oil through oral gavage)	27.43 ± 0.24	42.52 ± 0.68	40.80
2.	Group II: Disease control (DC) (DMBA 1 mg/kg through oral gavage)	30.27 ± 0.52	39.13 ± 0.74^**^	37.23^**^
3.	Group III: Treatment (HIS 10 mg/kg through i. p.)	35.18 ± 0.21	41.08 ± 0.59^*^	38.88^*^
4.	Group IV: Treatment (HIS-CMC NPs 10 mg/kg through i. p.)	37.01 ± 0.36	43.62 ± 0.63^**^	41.31^**^
5.	Group V: Positive control (Cisplatin 5 mg/kg through i. p.)	26.23 ± 0.53	35.46 ± 0.71^**^	33.82^**^

Data is represented as Mean ± SD (n=6). Statistically significant differences were indicated as (*p < 0.05 and **p < 0.01) for the treated groups compared with the disease control (DC) group.

#### 3.3.2 Estimation of haematological and biochemical parameters

DMBA-induced mammary carcinogenesis significantly altered the haematological and biochemical profile of the animals (**Table 2**). Carcinogenesis-associated inflammation and anaemia were indicated by the disease control group (Group II) due to a marked increase in total WBC count and platelet count, in addition to a substantial decrease in RBC count and haemoglobin levels as compared to the normal control (Group I). These changes were successfully reversed in HIS treated (Group III) and HIS-CMC NPs treated (Group IV), with NPs demonstrating higher efficacy by dramatically reducing increased WBCs and platelets while reducing RBC and haemoglobin levels to nearly normal levels. The cisplatin treated group (Group V) also showed improvement in several haematological and biochemical parameters compared with the DMBA-induced group (Group II). However, mild alterations in hepatic and renal markers were still observed, consistent with the known toxicity of cisplatin. In group II, significant hepatic and renal deterioration was also shown by serum biochemical markers. DMBA-induced hepatotoxicity was confirmed by elevated levels of total bilirubin, ALP, SGPT and SGOT in addition to decreased levels of total protein and albumin. These parameters were improved by both treatments; however, HIS-CMC NPs had a stronger hepatoprotective impact than free HIS, bringing bilirubin, protein, and enzyme levels to normal. Group II also had slightly higher levels of creatinine and urea, but both treatments kept these renal markers below the normal range, suggesting nephroprotective potential. DMBA considerably raised total cholesterol and decreased HDL levels, which were successfully altered by treatment with HIS and HIS-CMC NPs, with HIS-CMC NPs exhibiting the most lipid-regulating impact by significantly lowering total cholesterol and raising HDL levels. While several tests indicated non-significant differences, most of the haematological, hepatic, renal, and lipid parameters in the group III and group IV showed substantial improvements (*p* < 0.001) in comparison to the group II.

**Table 2:** Haematological and biochemical parameters of experimental animals.

S. no.	Investigation	Group I	Group II	Group III	Group IV	Group V
Haematological parameters						
1.	Total WBC count (×10^3^/µL)	7.2 ± 0.8	15.6 ± 1.5	7.8 ± 0.9^***^	6.1 ± 0.4^***^	5.1 ± 0.6^***^
2.	RBC count (×10^6^/µL)	8.32 ± 0.4	6.41 ± 0.5	7.36 ± 0.3^**^	^†^7.95 ± 0.4^***^	4.89 ± 0.5^***^
3.	Haemoglobin (%)	13.81 ± 0.6	10.23 ± 0.8	11.82 ± 0.6^***^	^†^12.15 ± 0.5^***^	9.67 ± 0.7^#^
4.	Platelet count (×10^3^/µL)	900 ± 80	1267 ± 91	984 ± 83^***^	^†^918 ± 76^***^	552 ± 64^***^
Liver function parameters						
5.	Total bilirubin (mg/dL)	0.31 ± 0.1	0.54 ± 0.1	0.38 ± 0.1^***^	^†^0.31 ± 0.1^***^	0.53 ± 0.1^#^
6.	Total protein (g/dL)	6.99 ± 0.3	5.58 ± 0.4	5.72 ± 0.3^#^	^†^6.34 ± 0.2^***^	5.11 ± 0.4^**^
7.	Albumin (g/dL)	3.71 ± 0.2	2.39 ± 0.3	2.67 ± 0.2^**^	^†^3.12 ± 0.2^***^	2.28 ± 0.3^***^
8.	SGOT (U/L)	60 ± 10	180 ± 15	95 ± 12^***^	^†^83 ± 10^***^	140 ± 20^***^
9.	SGPT (U/L)	45 ± 6	120 ± 10	60 ± 8^***^	^†^48 ± 6^***^	90 ± 12^***^
10.	Alkaline phosphatase (U/L)	85 ± 10	160 ± 15	110 ± 12^***^	^†^95 ± 10^***^	141 ± 15^***^
Renal function parameters						
11.	Creatinine (mg/dL)	0.30 ± 0.04	0.33 ± 0.05	0.31 ± 0.04^#^	0.30 ± 0.04^#^	0.65 ± 0.1^***^
12.	Urea (mgs/dL)	22 ± 3	33 ± 4	25 ± 3^***^	^†^24 ± 3^***^	45 ± 5^***^
Lipid parameters						
13.	Total cholesterol (mg/dL)	95.12 ± 0.6	160.46 ± 0.7	122.74 ± 0.4^***^	^†^107.41 ± 0.6^***^	112.13 ± 0.4^***^
14.	HDL cholesterol (mg/dL)	63.23 ± 0.5	38.11 ± 0.2	52.25 ± 0.4^***^	^†^56.32 ± 0.5^***^	51.54 ± 0.4^**^

Data is represented as Mean ± SD (n=6). Statistically significant differences were indicated as (^**^p< 0.01, ^***^p< 0.001 and ^#^ns – non-significant) for the treated groups compared with the DC group. ^†^indicate the values of group IV (HIS-CMC NPs) were near to the untreated control (Group I) indicating the restoration of DMBA-induced anomalies.

The HIS-CMC NPs showed a near to normal restorative values better than free HIS when compared to normal control (Group I) for parameters; body weight, inflammatory markers (WBC count), haemoglobin, hepatic enzymes (SGOT and SGPT), and lipid profile parameters (total cholesterol and HDL). This enhanced efficacy may be attributed to improved stability, bioavailability, sustained release and tissue distribution of HIS following CMC encapsulation. HIS-CMC NPs treatment demonstrated near to normal restoration of DMBA-induced alterations.

### 3.4 Histopathological evaluation

**Figure 4:**
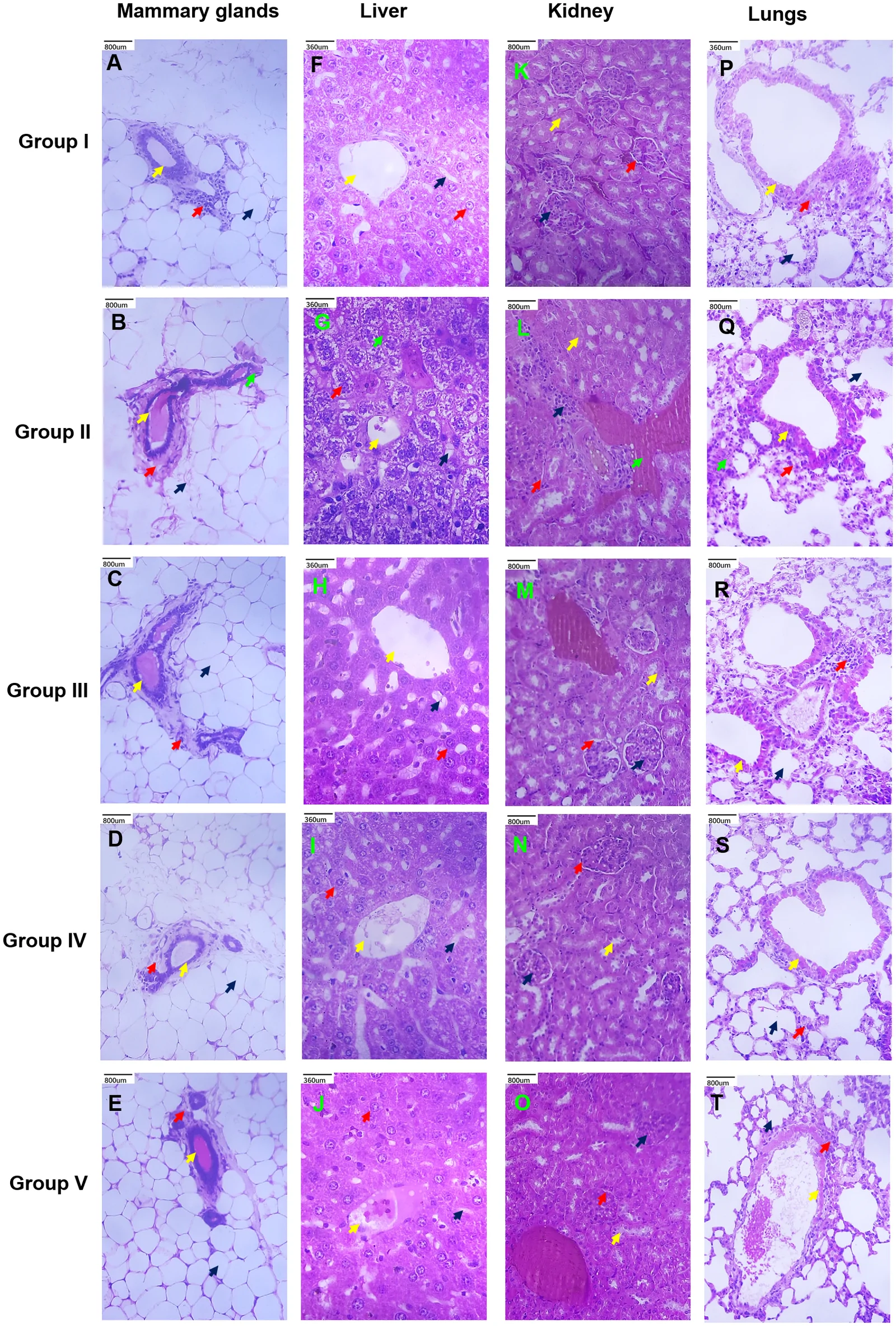
Histopathological analysis of animal tissue samples, **(A–E)** mammary glands,**(F–J)** liver, **(K–O)** kidney and **(P–T)** lung tissues of experimental animal groups showing various histological changes at 10X magnification (mammary glands, kidney and lungs); 20X magnification (liver).

The experimental groups consisted of Group I (normal control), Group II (DMBA-induced disease control), Group III (HIS), Group IV (HIS-CMC NPs), and Group V (Cisplatin). The mammary glands, liver, kidney and lungs of the experimental animals were investigated for the histological changes after the treatment. In the normal control (Group I) (**Figure 4A**), the mammary gland tissue displayed normal adipocyte structure (blue arrow) with intact ducts (yellow arrow) and with normal stroma and no indications of cellular atypia or inflammation (red arrow). Significant pathological alterations were seen in disease control (Group II) (**Figure 4B**), including enhanced periductal fibrotic reaction (blue arrow), stromal inflammation (red arrow), and dilated ducts with epithelial hyperplasia (yellow arrow). Occasional tumour-associated structures were also visible with the occurrence of congested blood vessels (green arrow). The mammary gland structure was partially altered in the HIS treated (Group III) animals (**Figure 4C**), but there was a clear significant decrease in inflammatory infiltration of stroma (red arrow), near normal adipocytes (blue arrow), and hyperplasia (yellow arrow), showing normal ducts as compared to the group II. The anti-tumour activity was demonstrated by HIS-CMC NPs treated (Group IV) (**Figure 4D**), which displayed normal architecture with minimum epithelial thickening (yellow arrow) and significantly reduced inflammatory signals in stroma and normal adipocytes (red and blue arrows). The animals treated with cisplatin (Group V) (**Figure 4E**) showed persistent stromal damage (red arrow) and considerable ductal hyperplasia (yellow arrow), but with some improvement compared to group II. Liver sections from the control group (**Figure 4F**) showed normal hepatocytes arranged in cords (red arrow) with clear sinusoids (blue arrow) and intact central veins (yellow arrow). The group II (**Figure 4G**) showed signs of sinusoidal dilatation (blue arrow), congested central vein (yellow arrow), hepatocellular degeneration and vacuolization (red arrow) and inflammatory infiltration (green arrow), all of which indicated systemic toxicity linked to tumour burden. The hepatic architecture was moderately restored in the group III (**Figure 4H**), with fewer degenerative hepatocytes (red arrow), decreased inflammation (blue arrow) and a near-normal central vein (yellow arrow). The HIS-CMC NPs treatment (**Figure 4I**) displayed substantial hepatoprotection, reflected by nearly normal hepatocyte morphology (red arrow), reduced cellular degeneration of sinusoids (blue arrow), and minimal inflammatory infiltration of the central vein (yellow arrow). Liver sections treated with cisplatin (**Figure 4J**) had moderate necrosis with sinusoidal inflammation (blue arrow), hepatocellular enlargement (red arrow) and prolonged inflammation in the central vein (yellow arrow), suggesting both drug-related hepatotoxicity and a partial recovery.

The histological analysis of kidney tissue in the control animals (**Figure 4K**) showed normal glomerular structures (blue arrow), intact Bowman’s capsule (red arrow) and defined tubules (yellow arrow). The significant tubular necrosis (yellow arrow), endocapillary proliferation in the glomeruli (blue arrow) and obliteration of the Bowman’s capsule with an inflammatory cell buildup (red arrow) were observed in the group II (**Figure 4L**). Interstitial oedema and congested blood vessels were also evident (green arrow). In group III (**Figure 4M**), moderate inflammatory alterations in glomeruli (blue arrow) with partial structural recovery, including decreased tubular necrosis (yellow arrow) and restoration of glomerular structure with near-normal Bowman’s capsule (red arrow), compared to group II, were observed. Group IV animals (**Figure 4N**) exhibited significant renal protection, with well-preserved glomeruli (blue arrow), minimum tubular injury with reduced inflammatory infiltration (yellow arrow) and intact Bowman’s capsule (red arrow) compared to group III. The cisplatin-induced nephrotoxicity was observed (**Figure 4O**), the group V showing significant tubular damage and glomerular shrinkage with inflammation (yellow and blue arrows), as well as unorganised Bowman’s capsule (blue arrow). The lung tissue of the control group (**Figure 4P**) showed normal interstitium (red arrow), intact bronchioles (yellow arrow) and normal alveolar spaces (blue arrow) with no inflammatory cells. In group II (**Figure 4Q**), the serious pathological alterations were observed, such as bronchial congestion (yellow arrow), thick and unorganised alveolar spaces (blue arrow), inflammatory cell infiltration of interstitium (red arrow) and tumour-associated structural deformation (green arrow). There was moderate improvement in the group III lungs (**Figure 4R**) compared to the group II, with reduced inflammation in the interstitium (red arrow), near-normal alveolar spaces and partial normalisation of bronchial thickness (blue and yellow arrows). Significant pathological alterations were reversed in the group IV (**Figure 4S**), with normal bronchial morphology (yellow arrow), reduced inflammatory infiltration (red arrow) and normal alveolar spaces (blue arrow). The cisplatin-treated lungs (**Figure 4T**) showed improvement compared with the group II but retained areas of congestion in bronchi (yellow arrow), inflammatory infiltration (red arrow) and unorganised alveolar spaces (blue arrow), consistent with known pulmonary toxicity.

The comparative histopathological evaluation demonstrated that HIS-CMC NPs displayed better protection against DMBA-induced tissue damage than free HIS. The NPs consistently preserved normal tissue architecture and minimised pathological alterations across the mammary gland, liver, kidney and lungs to a greater extent than the free drug. These findings indicate that CMC-mediated encapsulation enhanced the tissue-protective effects of hispidulin, resulting in improved attenuation of inflammation and cellular injury. The histological outcomes observed with HIS-CMC NPs further support the enhanced therapeutic efficacy of the nanoparticle formulation.

### 3.5 Immunohistochemical evaluation of *Ki67* and *Nrf2* expression

**Figure 5:**
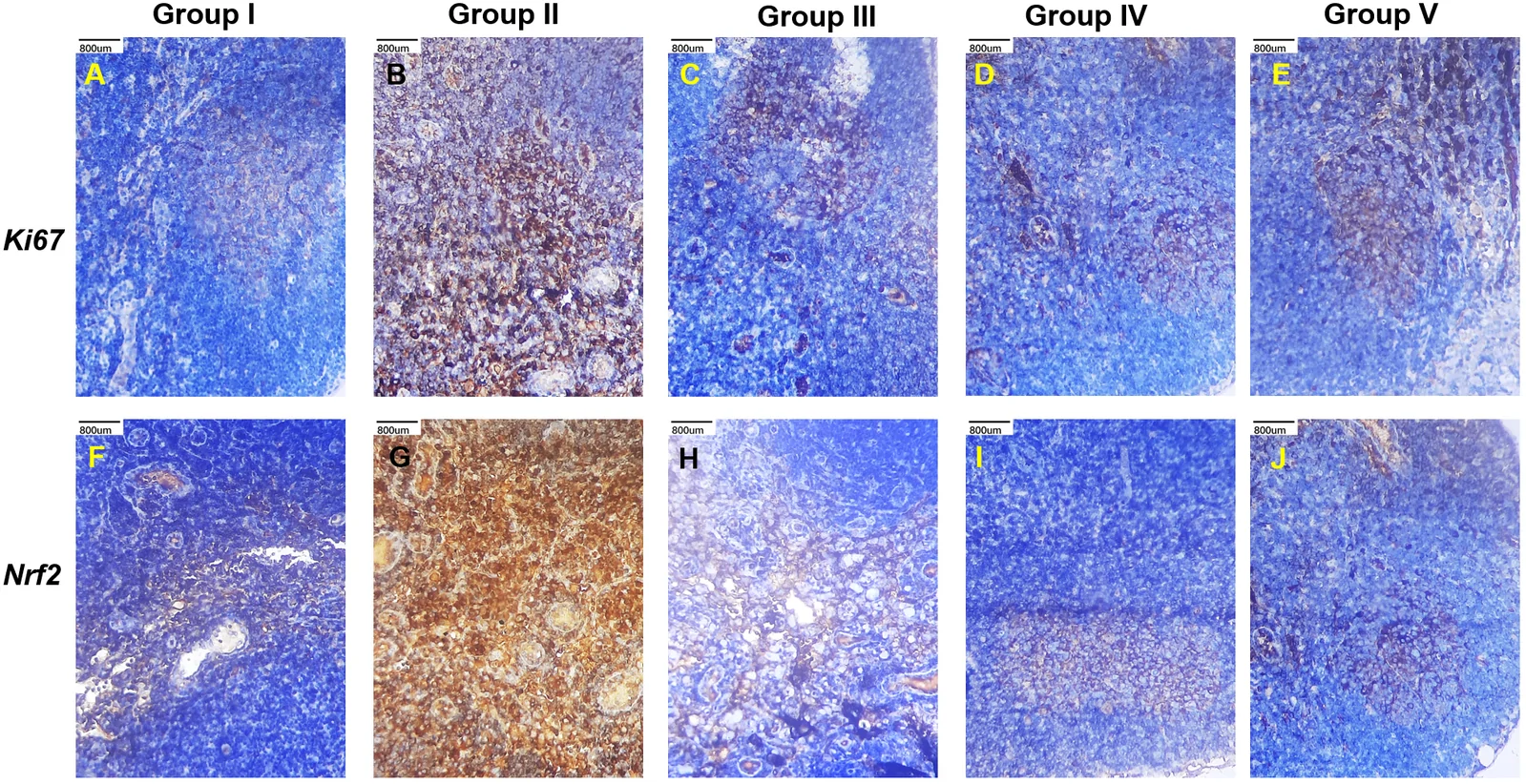
Immunohistochemistry of mammary gland tissues across experimental groups showing *Ki67* **(A–E)** and *Nrf2* **(F–J)** expression in groups (I, II, III and IV), highlighting differences in proliferative and oxidative stress markers at 10X magnification.

The immunohistochemistry analysis revealed marked differences in *Ki67* and *Nrf2* expression among the experimental groups. In the normal control (Group I), *Ki67* expression was minimal, reflecting normal mammary gland architecture with low basal proliferative activity (**Figure 5A**). In the same manner, *Nrf2* staining was weak and evenly distributed, suggesting adequate physiological antioxidant regulation in the absence of oxidative stress (**Figure 5F**). The disease control (Group II) showed strong proliferative activity typical for DMBA-induced mammary carcinogenesis, as evidenced by the extensive and intense *Ki67* positivity (**Figure 5B**). Similarly, there was a significant upregulation of *Nrf2* expression, which indicated increased oxidative stress and the activation of compensatory antioxidant mechanisms (**Figure 5G**). The HIS treatment (Group III) resulted in a notable decrease in *Nrf2* immunoreactivity (**Figure 5H**) and *Ki67*-positive nuclei (**Figure 5C**), indicating attenuation of oxidative stress and abnormal cell growth. HIS-CMC NPs treated (Group IV) had demonstrated a significant decrease in both markers (**Figure 5D** and I), approaching levels observed in the control group. This implies that the nanoparticle formulation has better anti-proliferative and antioxidant activity. In cisplatin treated (Group V), *Ki67* and *Nrf2* expression levels (**Figures 5E, J**) were lower than those observed in group II. However, oxidative stress and proliferative activity were still evident in several tissue areas. Comparative immunohistochemical observations further substantiated the enhanced therapeutic efficacy of HIS-CMC NPs over free HIS. The nanoparticle formulation produced a significant decrease in *Ki67* and *Nrf2* immunoreactivity, indicating more effective suppression of abnormal cellular proliferation and oxidative stress. These findings are consistent with the improved histopathological preservation observed in the HIS-CMC NP-treated group and suggest that CMC encapsulation enhanced the biological activity of hispidulin. Collectively, the immunohistochemical results reinforce increased anticancer and antioxidant potential of the NPs. Overall, the IHC findings indicate that HIS-CMC NPs exerted inhibitory effect on cancerous cell proliferation and oxidative stress pathways than free hispidulin or cisplatin, correlating with the improved histopathological outcomes observed.

### 3.6 Estimation of antioxidant parameters

#### 3.6.1 Malondialdehyde activity

**Figure 6:**
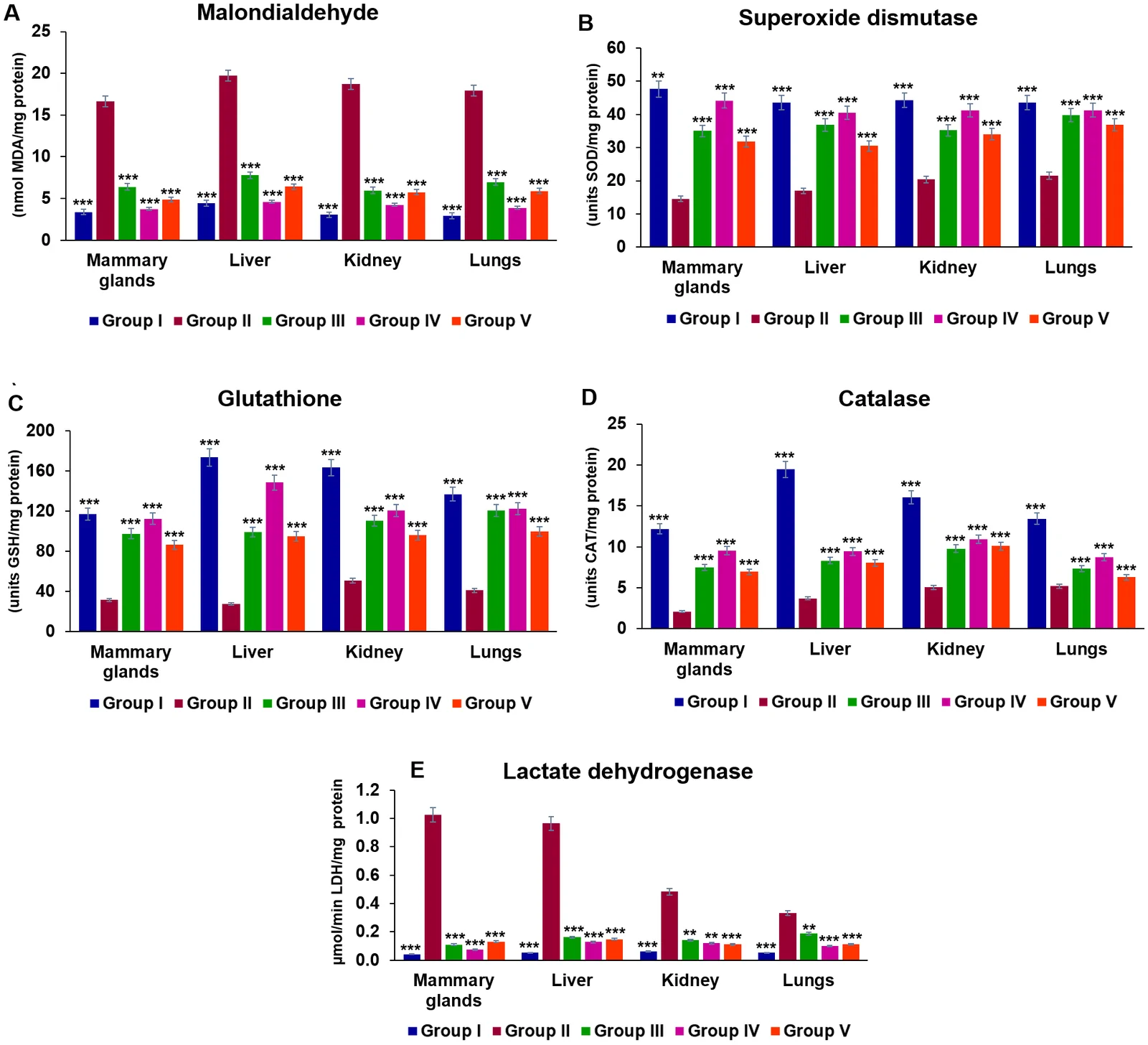
Biochemical parameters in groups (I, II, III, IV and V) showing **(A)** lipid peroxidation (MDA), **(B)** superoxide dismutase (SOD), **(C)** reduced glutathione (GSH), **(D)** catalase (CAT) and **(E)** lactate dehydrogenase (LDH) levels measured in various tissues. Data are presented as mean ± SEM (n = 6), where ***p < 0.001 indicates statistical significance compared to group II.

Malondialdehyde (MDA) is an important byproduct of lipid peroxidation, and its level is widely used as a sensitive biochemical marker to assess the extent of oxidative stress-induced damage to cellular and tissue membranes. In comparison to normal control (Group I), disease control (Group II) had significantly higher levels of malondialdehyde (MDA) in all tissues analysed, indicating significant DMBA-induced lipid peroxidation observed (**Figure 6A**). On the other hand, compared to group II, HIS treated (Group III), HIS-CMC NPs treated (Group IV), and cisplatin treated (Group V) significantly reduced MDA levels in all tissues, indicating efficient attenuation of oxidative stress. The significantly reduced MDA content was observed for group IV. In the mammary gland, group II animals had the highest MDA levels, while group III, group IV and group V significantly reduced MDA toward near-normal values. In the same way, liver MDA was increased in group II but significantly lowered with treatments. Increased MDA in group II was also seen in lung and kidney tissues, indicating systemic oxidative damage. Thus, HIS-CMC NPs substantially restored MDA levels to control values (*p* < 0.001), and more effectively normalised lipid peroxidation than free HIS (**Table 3**).

**Table 4:** Effect of HIS and HIS-CMC NPs on biochemical parameters in the tissues (mammary glands, liver, kidney and lungs) of DMBA induced BALB/c mice.

S. no.	Biochemical assays	Group	Mammary gland	Liver	Kidney	Lungs
1.	MDA (nmol/MDA/mg protein)	I	3.38 ± 0.05^#^	4.42 ± 0.22^#^	3.03 ± 0.07^#^	2.91 ± 0.32^#^
		II	16.67 ± 0.17	19.74 ± 0.27	18.71 ± 0.80	17.95 ± 0.09
		III	6.39 ± 0.10^#^	7.77 ± 0.05^#^	5.95 ± 0.25^#^	6.96 ± 0.37^#^
		IV	^†^4.86 ± 0.10^#^	6.41 ± 0.09^#^	5.73 ± 0.21^#^	5.87 ± 0.21^#^
		V	3.73 ± 0.16^#^	4.60 ± 0.14^#^	4.24 ± 0.29^#^	3.88 ± 0.57^#^
2.	SOD (units SOD/mg protein)	I	47.66 ± 0.18^@^	43.60 ± 0.90^#^	44.28 ± 1.22^#^	43.62 ± 1.92^#^
		II	14.59 ± 2.13	16.96 ± 0.99	20.43 ± 1.61	21.57 ± 2.15
		III	35.02 ± 2.71^#^	36.82 ± 2.75^#^	35.20 ± 0.71^#^	39.84 ± 0.10^#^
		IV	^†^44.20 ± 1.25^#^	^†^40.55 ± 1.93^#^	^†^41.23 ± 2.41^#^	^†^41.27 ± 0.16^#^
		V	31.81 ± 1.71^#^	30.49 ± 1.12^#^	34.06 ± 1.02^#^	36.85 ± 0.09^#^
3.	GSH (units GSH/mg protein)	I	116.89 ± 0.07^#^	173.49 ± 0.04^#^	163.33 ± 0.06^#^	136.89 ± 0.03^#^
		II	31.53 ± 0.01	27.49 ± 0.08	50.86 ± 0.04	41.21 ± 0.03
		III	97.57 ± 0.03^#^	99.12 ± 0.05^#^	110.38 ± 0.05^#^	120.75 ± 0.02^#^
		IV	^†^112.31 ± 0.01^#^	^†^148.40 ± 0.03^#^	^†^120.70 ± 0.05^#^	^†^122.34 ± 0.02^#^
		V	86.29 ± 0.01^#^	94.81 ± 0.07^#^	95.98 ± 0.05^#^	99.69 ± 0.06^#^
4.	CAT (units CAT/mg protein)	I	12.19 ± 0.07^#^	19.47 ± 0.01^#^	16.06 ± 0.03^#^	13.45 ± 0.03^#^
		II	2.09 ± 0.03	3.72 ± 0.07	5.06 ± 0.12	5.21 ± 0.03
		III	7.47 ± 0.03^#^	8.31 ± 0.07^#^	9.79 ± 0.06^#^	7.32 ± 0.03^#^
		IV	^†^9.54 ± 0.05^#^	9.43 ± 0.04^#^	^†^10.93 ± 0.03^#^	8.74 ± 0.05^#^
		V	6.95 ± 0.04^#^	8.05 ± 0.06^#^	10.09 ± 0.04^#^	6.29 ± 0.05^#^
5.	LDH (µmol/min LDH/mg protein)	I	0.043 ± 0.02^#^	0.053 ± 0.01^#^	0.062 ± 0.01^#^	0.054 ± 0.03^#^
		II	1.027 ± 0.04	0.966 ± 0.02	0.484 ± 0.10	0.334 ± 0.31
		III	0.110 ± 0.01^#^	0.162 ± 0.03^#^	0.141 ± 0.08^@^	0.188 ± 0.02^@^
		IV	0.075 ± 0.01^#^	0.128 ± 0.01^#^	0.121 ± 0.07^#^	^†^0.101 ± 0.06^#^
		V	0.132 ± 0.03^#^	0.149 ± 0.03^#^	0.112 ± 0.02^#^	0.113 ± 0.05^#^

Data is represented as Mean ± SD (n=6). Statistically significant differences were indicated as (**p < 0.01 denoted by ^@^ and ***p < 0.001 denoted by ^#^) for the groups (II, III, IV and V) compared with the disease control (II) group. † indicate the value of Group IV is either better than Group III or values approaching to near normal compared with normal control (Group I).

#### 3.6.2 Superoxide dismutase activity

Superoxide dismutase (SOD) is a key antioxidant enzyme that protects tissues from oxidative damage by converting superoxide radicals into less harmful molecules. Improved cellular defence systems and less oxidative stress are typically associated with higher SOD activity. In the present study, SOD activity was significantly altered among the experimental groups in all examined tissues (**Figure 6B**). The disease control (Group II) demonstrated a marked and significant decline in SOD activity compared to the normal control (Group I). The SOD levels were significantly lower in group II than in group I, indicating that DMBA treatment had caused oxidative stress. SOD activity was considerably recovered in HIS treated (Group III) and HIS-CMC NPs treated (Group IV), with the nanoparticle formulation exhibiting the most noticeable impact. Group III displayed higher activity than group II. Cisplatin treatment (Group V) also improved the antioxidant status. Notably, the group IV showed a significant increase in SOD activity. In all tissues, the SOD activity of the group II was considerably lower than that of the group I (p< 0.001), indicating severe oxidative stress caused by DMBA. SOD levels were significantly recovered in a tissue-dependent manner by HIS-CMC NPs compared to HIS demonstrating the significant improvement (*p* < 0.001).

#### 3.6.3 Glutathione reductase activity

Glutathione reductase is a key antioxidant enzyme that maintains cellular redox balance by regenerating reduced glutathione (GSH) from its oxidised form. Enhanced detoxifying capacity and improved tissue resistance to oxidative injury are indicated by increased activity of this enzyme. Disease control (Group II) showed a significant decrease in GSH content, indicating significant depletion of antioxidant responses due to DMBA-induced oxidative stress, in contrast to normal control (Group I), which displayed high GSH levels (**Figure 6C**). While cisplatin treatment (Group V) and HIS treated (Group III) displayed an increased GSH activity. Notably, HIS-CMC NPs treated (Group IV) demonstrated the highest GSH levels, indicating superior antioxidant defence and effective attenuation of DMBA-mediated glutathione depletion. The glutathione reductase activity in group II was significantly lower than that of group I (*p* < 0.001). Treatment with HIS and HIS-CMC NPs resulted in a significant enzyme activity (**Table 3**), with HIS-CMC NPs exhibiting the most notable improvement (*p<* 0.001) in comparison with HIS.

#### 3.6.4 Catalase activity

Catalase is a key enzyme that helps neutralise harmful hydrogen peroxide in cells. A higher level of catalase activity suggests improved defence against oxidative damage. A higher level of catalase activity suggests improved defence against oxidative damage. Disease control (Group II) demonstrated a marked decrease in CAT activity, indicating a significant impairment of antioxidant defences after DMBA exposure compared to normal control (Group I) (**Figure 6D**). The cisplatin treatment (Group V) resulted in a slight improvement in CAT activity, while treatment with HIS (Group III) significantly increased the activity. The HIS-CMC NPs treatment (Group IV) showed the highest catalase activity, indicating superior enhancement of antioxidant capacity and effective mitigation of DMBA-induced catalase depletion. Thus, the treatment with HIS and HIS-CMC NPs produced a significant elevation in enzyme levels (**Table 3**), with HIS-CMC displaying enhanced activity (*p* < 0.001) compared to HIS.

#### 3.6.5 Lactate dehydrogenase activity

Lactate dehydrogenase (LDH) is a cellular enzyme that is released into tissues when cells are damaged or undergo membrane disruption. Increased cellular damage and metabolic stress are usually indicated by elevated LDH activity. Disease control (Group II) showed a significant increase in LDH activity compared to normal control (Group I) (**Figure 6E**). This indicates significant cellular damage and LDH leakage due to DMBA-induced toxicity. LDH activity was significantly reduced in HIS treated (Group III) than in Group II. The comparable decrease was obtained by cisplatin treatment (Group V). The HIS-CMC NPs treatment (Group IV) showed a noticeable decrease in LDH activity, suggesting better protection against membrane damage and efficient reduction of DMBA-induced cytotoxicity compared to HIS (**Table 3**). While treatment with HIS and HIS-CMC NPs resulted in a significant reduction of LDH levels, with HIS-CMC exhibiting the strongest protective effect (*p* < 0.001) than HIS, the LDH activity was significantly higher in group II compared to group I (*p* < 0.001).

The findings demonstrate that both free hispidulin (Group III) and HIS-CMC nanoparticles (Group IV) attenuated DMBA-induced oxidative stress by restoring endogenous antioxidant defence and reducing oxidative damage. However, the HIS-CMC nanoparticle formulation exhibited restoration of SOD, GSH, and CAT activities, along with a better reduction in MDA and LDH levels, compared with free hispidulin. These observations suggest that encapsulation of hispidulin within CMC nanoparticles enhanced its antioxidant potential through improved bioavailability, sustained drug release, and efficient cellular uptake. Consequently, the restoration of the antioxidant defence system by HIS-CMC nanoparticles may have contributed to improved protection against oxidative stress-mediated tissue damage and the overall chemopreventive efficacy observed in the DMBA-induced mammary carcinogenesis model.

## 4 Discussion

The therapeutic potential of HIS-CMC NPs and their increased anticancer efficacy against MCF-7 cells was demonstrated in this work. The higher bioavailability, prolonged release and better cellular absorption of the encapsulated flavonoid in the HIS-CMC NPs showed better anticancer activity than free HIS. The *in vitro* evaluation served as a critical initial step, enabling a detailed assessment of cytotoxicity, apoptotic response and intracellular oxidative stress under controlled conditions. The sulforhodamine B assay is a cost-effective colourimetric method widely used to assess cancer cell proliferation and cytotoxicity by measuring total cellular protein content. It is an ideal method for assessing the impact of drugs on cell proliferation since it stains proteins and the resulting absorbance precisely correlates with cell quantity. The scalability, sensitivity and repeatability in drug screening across different cancer cell lines are determined through this assay (50). In the SRB assay, both HIS and HIS-CMC NPs in the present study showed strong antiproliferative effects on MCF-7 cells in a concentration-dependent manner. The higher inhibitory action of the NPs formulation is supported by the lower IC_50_ value of HIS-CMC NPs. This finding is consistent with the literature, where nanoparticle drug delivery systems improved efficacy and reduced IC_50_ values compared to free drugs. The higher cytotoxicity of HIS-CMC in comparison to free HIS indicated improved cellular uptake, HIS degradation protection, and prolonged intracellular release by the nanocarrier system, all of which contributed to the cytotoxic effect.

The DCFH-DA is a widely used fluorescence-based technique assessing intracellular reactive oxygen species (ROS) in cancer cells. In this assay, non-fluorescent DCFH-DA diffuses into cells, where esterases deacetylate and convert it to DCFH, which is subsequently oxidised by ROS to the highly fluorescent DCF. As a result, the increase in green fluorescence is proportional to cellular ROS levels and represents oxidative stress brought by anticancer treatments (51). In the present study, the nanoencapsulation of HIS in CMC promotes oxidative stress-mediated cytotoxicity in MCF-7 cells. The increased ROS levels seen in HIS-CMC-treated cells most likely result from more sustained HIS release and better intracellular delivery, which causes oxidative damage.

DAPI assay is a fluorescence-based technique for visualising cell nuclei and identifying nuclear alterations linked to cancer cell apoptosis. DAPI (4′,6-diamidino-2-phenylindole) binds to the A-T-rich sections of DNA and generates brilliant blue fluorescence under UV light, allowing assessment of chromatin condensation and nuclear fragmentation that signal apoptotic cell death (52). The present study demonstrates that HIS promotes apoptosis in MCF-7 cells as evidenced by chromatin condensation and nuclear fragmentation apparent with DAPI staining. A greater apoptotic index and more noticeable nuclear alterations demonstrated that the pro-apoptotic potency of HIS is further enhanced by nanoencapsulation in CMC. This increase in apoptosis correlates with prior data demonstrating greater reactive oxygen species formation and lower viability in HIS-CMC NPs treatments, confirming a mechanism where oxidative stress promotes apoptotic cell death.

The AO/EB staining assay is a simple fluorescent method for detecting apoptosis in cancer cells by observing live cells with green nuclei and apoptotic or dead cells with orange-red nuclei based on membrane integrity (53). The findings of the present study indicated that HIS induced apoptosis in MCF-7, with enhanced apoptotic efficacy seen upon nanoencapsulation in CMC NPs, as evidenced by increased AO/EB staining of apoptotic nuclei. The enhanced stability and sustained drug-release behaviour of HIS-CMC NPs increased production of ROS, and disorganisation of the mitochondrial membrane caused by NPs are the primary causes of the higher apoptotic index. These factors, in general, activate intrinsic apoptotic pathways more effectively compared to free drug reported in the other previous nanoparticles studies (54, 55).

The absence of cytotoxicity, significant ROS generation, nuclear damage, and apoptotic cell death observed following CMC treatment indicate that the polymer itself is biocompatible and does not exert any biological effects on MCF-7 cells. These findings suggest that the enhanced antiproliferative and pro-apoptotic activities observed with HIS-CMC NPs are primarily attributable to the encapsulated hispidulin rather than the CMC carrier, supporting the suitability of CMC as a safe drug delivery vehicle.

The *in-vivo* evaluation remains a crucial component of cancer research, as it provides physiologically relevant information on systemic toxicity, progression of carcinogenesis and therapeutic safety (56). In animal models, body weight and general development patterns are some of the most sensitive measures of treatment-related stress. Over the course of the 16-week study period, all experimental groups, Group I (normal control), Group II (DMBA-induced disease control), Group III (HIS), Group IV (HIS-CMC NPs), and Group V (Cisplatin), showed a steady increase in body weight and growth rate, associated with systemic toxicity. The HIS-CMC NPs demonstrated a sustained growth rate, showing a growth pattern similar to that of group I, even after extended treatment, indicating good physiological tolerance and biocompatibility. However, the animals treated with cisplatin had a slightly lower body weight. Thus, the drug administration reduced systemic toxicity by maintaining the normal growth patterns in DMBA-induced mammary carcinogenesis in mice. Increased levels of free radicals, enhanced lipid peroxidation and epithelial cell death could all have contributed to cellular membrane damage as a result of the DMBA dose interfering with normal biochemical processes in the body and reducing the function of antioxidant enzymes. As a result, these factors may have affected the total body weight as per the report based on previous nanoparticles study (57).

In the present study, no palpable or completely visible mammary tumours were detected in Groups II, III, IV and V. Despite the absence of macroscopic tumours, the histological analysis of Groups II, III, IV and V revealed distinct preneoplastic changes such as dysplasia and hyperplasia, before the treatment with HIS, HIS-CMC NPs and Cisplatin. In Group I, the mammary gland tissue was observed to be normal. Thus, the histopathological results obtained displayed the formation of precancerous lesions in the mammary gland tissue across the groups II, III, IV and V, confirming the carcinogenic effect of DMBA (58). Following confirmation of precancerous lesions in mammary gland tissues, animals from groups II, III, IV, and V were treated with HIS, HIS-CMC NPs, and cisplatin, respectively. In comparison to group II, group III and group IV significantly reduced the earlier observed microscopic alterations, suggesting a possible inhibitory effect on the course of mammary carcinogenesis under the treatment conditions.

The liver and kidneys are essential for detoxification and metabolic control; any disruption to their functions can have a significant impact on systemic physiology and increase toxicity (59). Based on the biochemical findings of the present study, significant liver damage was generated by DMBA exposure in this investigation, as shown by increased levels of total bilirubin, SGOT, SGPT and ALP, as well as decreased levels of total protein and albumin, which represent oxidative stress-mediated hepatocellular damage and impaired protein synthesis. These biochemical abnormalities were successfully restored by treatment with HIS and HIS-CMC NPs, demonstrating significant hepatoprotective benefits. Compared with free HIS, HIS-CMC NPs demonstrated greater restoration of the altered biochemical parameters, which may be attributed to their enhanced stability, sustained drug release, and improved cellular uptake, resulting in improved therapeutic efficacy.

Based on the lipid parameters, group II had significantly lower HDL levels and higher total cholesterol, suggesting a metabolic imbalance and increased lipid peroxidation associated with cancer. These anomalies were found to be significantly lowered in the HIS and HIS-CMC NPs treated groups. In comparison with free HIS, the nanoformulation restored lipid parameters closer to normal values, indicating enhanced therapeutic efficacy. HIS-CMC NPs’ potential to lessen tumour burden and systemic toxicity is supported by its superior control of lipid metabolism and oxidative stress, as seen by their ability to drop total cholesterol and raise HDL as per the other previous study based on lipoproteins (60). Increased blood urea and creatinine levels in group II indicated severe renal impairment brought on by DMBA treatment, most likely as a result of ROS-induced tubular and endothelial damage. Strong nephroprotective activity was demonstrated by the successful restoration of several kidney biomarkers following treatment with HIS and HIS-CMC NPs. The greater restoration observed with HIS-CMC NPs compared with free HIS further supports the therapeutic advantage of the nanoformulation. The enhanced bioavailability and prolonged release of HIS-CMC NPs may contribute to their better efficacy by improving antioxidant action and promoting the healing of injured renal tissues. Overall, the findings of this study indicate that HIS-CMC NPs effectively ameliorated DMBA-induced haematological, hepatic, renal and lipid alterations, demonstrating superior therapeutic performance compared to free HIS which is consistent with previous studies reported for other drug loaded biopolymeric nanocarriers (61–63).

The histopathological investigation of the current work shows that hispidulin successfully reduces carcinogen-induced histopathological changes in the mammary gland, liver, kidney and lung tissues in BALB/c mice, especially when administered through HIS-CMC NPs. Ductal hyperplasia, stromal inflammation, hepatocellular degeneration, renal tubular necrosis and pulmonary congestion were among the traditional signs of tumour growth and systemic toxicity seen in group II animals. These alterations shows the systemic oxidative stress and inflammatory response brought on by carcinogenesis which is consistent with the other previous study (64). The direct anticancer actions, along with the inhibition of tumour-associated inflammation, are probably responsible for the partial restoration of normal tissue architecture seen in group III based on the earlier study reported for compound hispidulin (65). Compared with free HIS, HIS-CMC NPs produced greater restoration of normal tissue architecture with reduced inflammatory changes, indicating enhanced therapeutic efficacy. The enhanced histopathological recovery observed with HIS-CMC NPs may be attributed to improved drug stability and sustained release which together contribute to greater cytoprotection and therapeutic efficacy than free HIS. These results corroborate earlier findings that by enhancing biodistribution and lowering metabolic breakdown, polymeric carriers can dramatically boost the anticancer effect of poorly soluble medicines (66). The animals treated with cisplatin showed some decrease in cancer cell proliferation but continued to exhibit severe organ toxicity. Thus, cisplatin causes intrinsic apoptosis in quickly proliferating cells by causing mitochondrial damage and DNA crosslinking. The prolonged necrosis, tubular damage, and pulmonary congestion seen in group V are consistent with previously documented profiles of cisplatin-induced organ toxicity (67, 68). Together, the data demonstrated that hispidulin, particularly when nano-formulated, mitigates carcinogen-induced damage by inhibiting cancerous cell growth, reducing oxidative stress and protecting normal tissues.

The immunohistochemical examination of mammary gland tissues in the present study revealed distinct expression patterns of *Ki67* and *Nrf2*, representing oxidative stress response and carcinogenesis activity across experimental groups. *Ki67* is a nuclear protein expressed in all active phases of the cell cycle but absent in quiescent cells, and it is widely used as a marker of tumour cell proliferation in mammary gland carcinogenesis. The elevated *Ki67* is commonly used to classify luminal tumours because it is associated with higher histological grade, a higher risk of recurrence and a lower chance of survival (69, 70). In this regard, the significant rise in *Ki67* in group II is indicative of the high mitotic activity of untreated precancerous mammary lesions, while its decrease in group III and group IV suggests that cell-cycle progression is suppressed and supports the antiproliferative effect of these treatments compared with the DMBA-induced group (Group II) as per the previous report of *Ki67* biomarker (71). The greater reduction in *Ki67* expression observed with HIS-CMC NPs compared with free HIS suggests an improved antiproliferative effect of the nanoformulation. *Nrf2* is a transcription factor that orchestrates the antioxidant and phase II detoxification response by inducing genes such as NQO1, HO-1, and glutathione-related enzymes, thereby maintaining redox homeostasis under stress. Prolonged *Nrf2* activation can function as a proto-oncogenic signal in breast cancer, encouraging migration, metabolic reprogramming, and proliferation (72, 73). Based on the present findings, both HIS and HIS-CMC NPs attenuated Nrf2 expression; however, the greater reduction observed in Group IV suggests improved restoration of redox homeostasis and enhanced therapeutic efficacy compared with free HIS. The strong *Nrf2* staining seen in the group II mammary glands is consistent with an adaptive response of rapid proliferation of cancer cells to oxidative stress as per the earlier studies of *Nrf2* biomarker (74, 75).

Based on the biochemical results of the present study, DMBA administration disturbed the redox homeostasis in the mammary gland, liver, kidney and lung tissues of the animals. This was indicated by increased levels of MDA and LDH activity, as well as decreased activities of important antioxidant enzymes (SOD, GSH and CAT). These alterations verify that DMBA causes oxidative stress and tissue damage, which supports the earlier findings showing effect of DMBA (76, 77). Additionally, the treatment with HIS and HIS-CMC NPs reversed these changes, lowering MDA and LDH levels and bringing SOD, GSH and CAT enzyme activities towards normal ranges. Compared with free HIS, HIS-CMC NPs produced greater restoration of these oxidative stress markers, indicating enhanced antioxidant efficacy. This strongly supports the antioxidant and cytoprotective role of hispidulin encapsulated in CMC. Hispidulin is known to have antioxidant properties by scavenging free radicals and preventing oxidative damage to biomolecules. It is more effective than traditional antioxidants in reducing lipid peroxidation and protecting protein thiol groups in some systems, according to the previous studies of compound hispiduin (78, 79). The enhanced antioxidant and cytoprotective effects of HIS-CMC NPs compared with free HIS may be attributed to the improved stability, sustained drug release, and enhanced bioavailability conferred by the nanoformulation. Other investigations using natural substances in DMBA-induced carcinogenesis models have found similar antioxidant-mediated chemopreventive effects. In hamsters with DMBA/croton oil-induced oral carcinogenesis, *Tamarix indica* extract effectively increased SOD and decreased glutathione levels (80). Similarly, in a rat model of DMBA-induced mammary cancer, treatment with *Terminalia chebula* extract enhanced SOD and catalase activity (81). Our results thus confirm that natural antioxidant molecules can successfully lessen oxidative damage brought on by exposure to carcinogens. These findings suggest that NPs enhances the therapeutic potential of natural antioxidants for cancer therapy.

**Figure 7:**
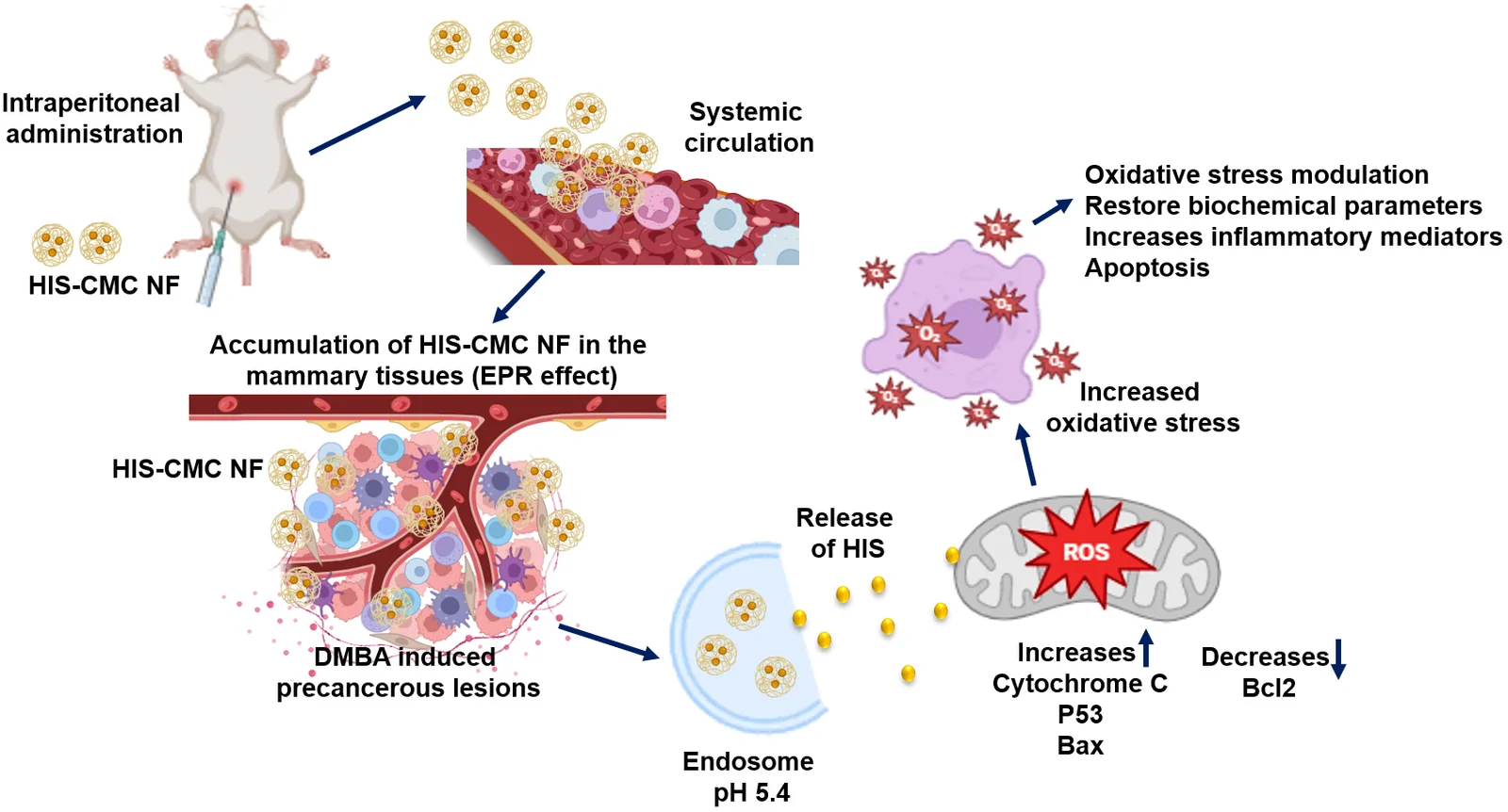
Proposed model depicting the chemopreventive effects of HIS-CMC NPs in mammary carcinogenesis *via* improved hispidulin delivery, redox modulation and activation of mitochondrial apoptosis.

Based on the *in vivo* findings, a hypothetical mechanism is proposed for the chemopreventive activity of HIS-CMC NPs in DMBA-induced mammary carcinogenesis (**Figure 7**). Following intraperitoneal administration, the nanoformulation may facilitate improved delivery of hispidulin owing to its physicochemical properties and sustained-release behaviour based on the earlier studies reported for nanomedicines (82). The CMC matrix provides sustained and pH-responsive release of hispidulin, which may enhance its bioavailability and therapeutic efficacy as per the previous study reported for CMC (83). Since mammary carcinogenesis is associated with oxidative stress, DNA damage and chronic inflammation, nanoencapsulated hispidulin may help restore redox homeostasis by modulating reactive oxygen species (ROS) and reducing oxidative damage. The sustained release and improved availability of nanoencapsulated hispidulin may further promote mitochondrial-mediated apoptosis through modulation of pro and anti-apoptotic signalling, leading to cytochrome c release and caspase activation. In addition, the formulation may suppress inflammatory and proliferative pathways, thereby limiting tumour progression as per the other study reported for hispidulin (41, 84). These combined effects may account for the improved histopathological architecture, restoration of biochemical parameters, and reduced systemic toxicity observed in treated animals. However, further molecular studies are required to validate the proposed mechanisms.

## 5 Conclusion

The findings of the present study demonstrate that hispidulin, particularly in its CMC-based NPs formulation, exhibits significant chemopreventive and therapeutic potential against DMBA-induced mammary carcinogenesis in BALB/c mice. *In vitro*, HIS-CMC NPs showed superior cytotoxicity toward MCF-7 cells, accompanied by enhanced ROS generation, pronounced nuclear damage, and markedly higher apoptotic induction compared with free HIS. Annexin-V/PI assay supported the significance of nanoencapsulation in enhancing the therapeutic efficacy and sustained release of the drug by demonstrating an efficient transition from early to late apoptosis. *In vivo*, treatment with HIS-CMC NPs effectively mitigated DMBA-mediated systemic toxicity, restoring haematological, hepatic, renal and lipid parameters toward normal physiological levels. The histopathological findings showed that group IV had nearly normal tissue architecture and few inflammatory or degenerative alterations in comparison to group II. The immunohistochemical analysis demonstrated that HIS-CMC NPs effectively suppressed aberrant proliferation and oxidative stress responses, as evidenced by greater reductions in *Ki67* and *Nrf2* expression. The nanoparticle formulation also showed strong antioxidant activity by dramatically lowering lipid peroxidation and LDH leakage while increasing endogenous antioxidant enzymes like SOD, GSH and catalase in mammary glands, liver, kidney and lung tissues through biochemical studies. Thus, HIS-CMC NPs represent a biocompatible nanoformulation with promising chemopreventive and therapeutic potential against mammary carcinogenesis. However, quantitative and qualitative observations (tumor weight and volume, survival etc), in the disease induced models and bio-distribution of free HIS versus HIS-CMC NPs in tumour tissue and major organs are the limitations of this study. Further studies are needed to elucidate the underlying molecular mechanisms and evaluate the long-term chemopreventive efficacy of HIS-CMC NPs before clinical application.

## Data Availability

The original contributions presented in the study are included in the article/**Supplementary Material**, further inquiries can be directed to the corresponding author/s.
