# Supplementary material for: Therapeutic efficacy of hispidulin-loaded carboxymethyl cellulose nanoparticles against mammary carcinogenesis through ROS-mediated apoptosis and tissue remodelling
Source: Front Oncol. 2026 Aug 20;16:1915182. doi: 10.3389/fonc.2026.1915182 (PMC13537956; doi:10.3389/fonc.2026.1915182)
Supplement: Supplementary file 1 [file Supplementaryfile1.docx]

**Supplementary Information**

**Figure S1.** UV-Vis spectrum of A) blank CMC showing characteristic absorption peak.

**Figure S2.** Representative SRB assay image of MCF-7 cells treated with blank CMC nanoparticles, demonstrating negligible cytotoxicity. Scale bar = 100 µm.

**Figure S3.** Fluorescence microscopic images of MCF-7 cells treated with blank CMC. A) DCFH-DA staining, B) DAPI staining, and C) AO/EB staining. Images were captured using a fluorescence microscope at 20x magnification with a scale bar of 100 µm.
